# Quantitative Imaging of the Action of vCPP2319, an Antimicrobial Peptide from a Viral Scaffold, against *Staphylococcus aureus* Biofilms of a Clinical Isolate

**DOI:** 10.1021/acsinfecdis.3c00195

**Published:** 2023-09-05

**Authors:** Susana A. Dias, Sandra N. Pinto, Ana S. Silva-Herdade, Marco Cavaco, Vera Neves, Luís Tavares, Manuela Oliveira, David Andreu, Ana Coutinho, Miguel A. R. B. Castanho, Ana Salomé Veiga

**Affiliations:** † Instituto de Medicina Molecular, Faculdade de Medicina, Universidade de Lisboa, Av. Prof. Egas Moniz, 1649-028 Lisboa, Portugal; ‡ iBB-Institute for Bioengineering and Biosciences and Associate Laboratory i4HB − Institute for Health and Bioeconomy at Department of Bioengineering, Instituto Superior Técnico, Universidade de Lisboa, Av. Rovisco Pais, 1049-001 Lisboa, Portugal; § CIISA − Centro de Investigação Interdisciplinar em Sanidade Animal, Faculdade de Medicina Veterinária, Universidade de Lisboa, Av. da Universidade Técnica, 1300-477 Lisboa, Portugal; ∥ Laboratório Associado para Ciência Animal e Veterinária (AL4AnimalS), 1300-477 Lisboa, Portugal; ⊥ Department of Medicine and Life Sciences, Pompeu Fabra University, Barcelona Biomedical Research Park, Dr. Aiguader 88, 08003 Barcelona, Spain; # Departamento de Química e Bioquímica, Faculdade de Ciências, Universidade de Lisboa, Campo Grande, 1749-016 Lisboa, Portugal

**Keywords:** bacterial biofilms, *Staphylococcus aureus*, antimicrobial peptides, combination therapy, bioimaging techniques

## Abstract

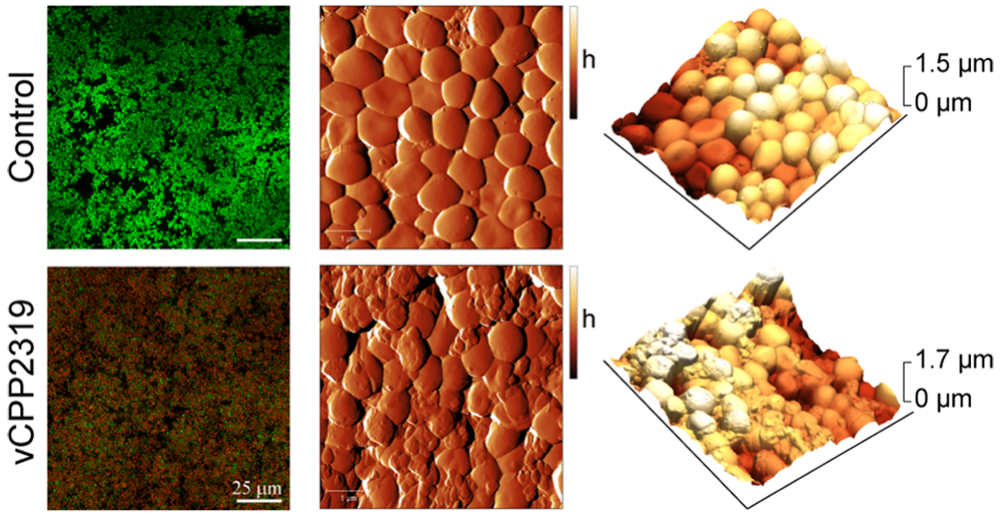

The formation of biofilms is a common virulence factor that makes bacterial infections difficult to treat and a major human health problem. Biofilms are bacterial communities embedded in a self-produced matrix of extracellular polymeric substances (EPS). In this work, we show that vCPP2319, a polycationic peptide derived from the capsid protein of Torque teno douroucouli virus, is active against preformed *Staphylococcus aureus* biofilms produced by both a reference strain and a clinical strain isolated from a diabetic foot infection, mainly by the killing of biofilm-embedded bacteria. The direct effect of vCPP2319 on bacterial cells was imaged using atomic force and confocal laser scanning microscopy, showing that the peptide induces morphological changes in bacterial cells and membrane disruption. Importantly, vCPP2319 exhibits low toxicity toward human cells and high stability in human serum. Since vCPP2319 has a limited effect on the biofilm EPS matrix itself, we explored a combined effect with α-amylase (EC 3.2.1.1), an EPS matrix-degrading enzyme. In fact, α-amylase decreases the density of *S. aureus* biofilms by 2.5-fold. Nonetheless, quantitative analysis of bioimaging data shows that vCPP2319 partially restores biofilm compactness after digestion of the polysaccharides, probably due to electrostatic cross-bridging of the matrix nucleic acids, which explains why α-amylase fails to improve the antibacterial action of the peptide.

Bacterial biofilms are well-organized communities of bacteria encased in a protective matrix^1,2^ composed mainly by self-produced extracellular polymeric substances (EPS), such as polysaccharides, proteins, and extracellular DNA (eDNA).^3^ Bacterial biofilms are responsible for most chronic and persistent infections in the human body, such as lung infections in cystic fibrosis or chronic wound infections, and are also frequent colonizers of biomedical devices.^4−6^ The Gram-positive bacterium *Staphylococcus aureus* (*S. aureus*), for instance, is an opportunistic pathogen with a high ability to form biofilms, associated with both device- and tissue-related infections.^7^ It is the most common species isolated from infected foot ulcers,^8,9^ a severe complication of diabetes mellitus^10,11^ and the most frequent cause of hospitalization of diabetic patients.^12,13^

Biofilm-related infections are difficult to treat because biofilm-embedded bacteria can evade host immune defenses and are less susceptible to conventional antibiotics than bacteria in the planktonic (“free floating”) form.^14,15^ The reduced diffusion, or even sequestration, of antibiotics in the biofilm EPS matrix contributes to the increased tolerance of the biofilms to antibiotics. To worsen matters, the dormancy of biofilm-embedded bacteria renders useless antibiotics that target metabolic processes. Antibiotic-based treatment of biofilms often fails despite prolonged use of high dosages. Thus, novel antimicrobial agents and strategies designed to target bacterial biofilms are urgently needed.

In recent years, antimicrobial peptides (AMPs) have emerged as promising candidates for antibiofilm agents^16−18^ based on their unique properties. Molecules of this class are present in all organisms as part of the host defense machinery, and are active against a wide range of microbial pathogens.^19,20^ AMPs usually have an overall cationic charge and a high content of hydrophobic amino acid residues, which allow them to target bacterial membranes that are anionic. Unlike conventional antibiotics, most AMPs kill bacteria fast upon contact with external membranes,^21−23^ hence bactericidal activity is independent of the metabolic status of target cells, giving AMPs the potential to kill slow-growing and nongrowing bacteria in biofilms. However, being polyelectrolytes, AMP diffusion through the biofilm matrix can be hindered due to charge interactions with the EPS components, slowing their availability at the inner layers of the biofilms.^24^ As such, the use of EPS matrix-degrading agents is a particularly attractive strategy to accelerate AMP penetration in biofilms,^25^ as a combination of molecules targeting biofilm components (EPS matrix and bacteria) is more likely to succeed in biofilm elimination than single-agent therapies.^26^

vCPP2319 is a cationic peptide reproducing residues 16–36 of the capsid protein of Torque teno douroucouli virus.^27^ It has cell-penetrating and anticancer properties,^27^ and it is also known for its broad-spectrum activity against bacteria in the planktonic form.^27,28^ Here, we investigated the activity of vCPP2319 against *S. aureus* biofilms formed by a reference strain and by a clinical strain isolated from a diabetic foot infection, of utmost medical importance.^8,9^ We used quantitative imaging methods to study the individual mechanism of action and the effect on biofilm structure of vCPP2319, and its combined effect with the matrix-degrading enzyme α-amylase from *Bacillus* sp. (EC 3.2.1.1), in search of an improved accessibility of the peptide to bacterial cells that would increase its activity.

## Results and Discussion

vCPP2319 is 20-residue, highly cationic peptide derived from the capsid protein of Torque teno douroucouli virus.^27^ Previous studies have demonstrated its broad-spectrum activity against both Gram-positive and -negative bacteria in the planktonic form, acting through the disruption of bacterial cell membranes.^27,28^ Here, we first focused on studying its serum stability and toxicity against human cells, and then on characterizing its activity against biofilms of *S. aureus*, a pathogen that is commonly involved in biofilm infections.^7^

### vCPP2319 Has High Biocompatibility and Stability

**Figure 1:**
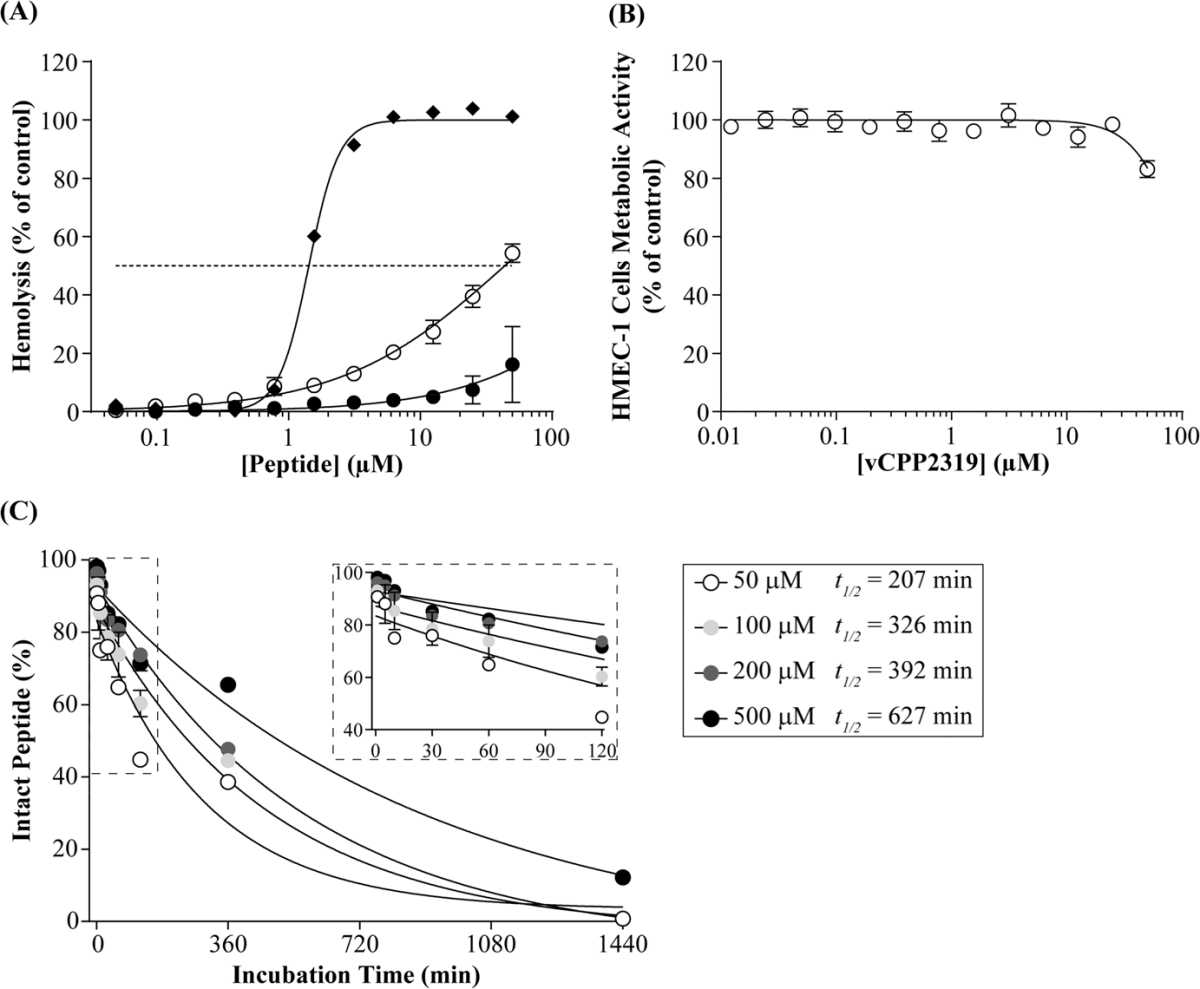
vCPP2319 toxicity and stability in human serum. (A) Hemolytic activity of vCPP2319, after incubation for 4 (●) or 24 h (○), and melittin (◆), a gold-standard for peptide cytotoxicity, after incubation for 1 h. (B) Cytotoxicity of vCPP2319 against HMEC-1 evaluated using the CellTiter-Blue cell viability assay after incubation for 24 h (○). (C) Kinetics of vCPP2319 degradation in human serum. Intact peptide fraction was calculated through chromatogram peak integration (Figure S1). The half-life (*t*_1/2_) was estimated by fitting the experimental data to a monoexponential decay model using GraphPad Prism version 7.0. The corresponding 95% confidence intervals (CI) are 139–401 for 50 μM, 234–542 for 100 μM, 327–490 for 200 μM, and 368–2107 for 500 μM.

We first evaluated the hemolytic effect of vCPP2319. Although the peptide has a mild effect on hRBCs, in a time- and concentration-dependent manner, it is clearly much less pronounced when compared with melittin, a gold-standard cytotoxic membrane-active peptide (Figure 1A). Thus, while melittin induces ca. 100% hemolysis at 3.13 μM after 1 h incubation, this value is only 13% for vCPP2319, at the same concentration, even after 24 h incubation. Considering the potential use of the peptide on wound infections, its toxicity was also tested using human dermal microvascular endothelium cells (HMEC-1 cells). As shown in Figure 1B, at concentrations of up to 25 μM, the peptide has no effect on the metabolic activity of these cells. Only at 50 μM, the highest concentration tested, a slight effect was observed, suggesting a low toxicity of vCPP2319 against dermal cells.

The stability of peptides is an important factor to take into account because their degradation in serum can reduce the concentration of the peptide and thus compromise its activity. As shown in Figure 1C (and Figure S1), for all vCPP2319 concentrations studied, after 1 h incubation with human serum, more than 60% of the peptide remains intact. The corresponding *t*_1/2_ values ranged from 207 min at 50 μM to 627 min at 500 μM, revealing high stability, even surpassing the *t*_1/2_ ∼ 112 min predicted by a software recently developed by us to estimate peptide half-lives.^29^ Interestingly, the stability of vCPP2319 increases with concentration, most likely due to the ability to self-assemble into nanoparticles (Figure S2) that protect the peptide from proteolytic degradation.^30,31^

### vCPP2319 Is Active against Preformed *S. aureus* Biofilms

The antibiofilm activity of vCPP2319 was tested against 24 h-preformed biofilms produced by two *S. aureus* strains: a reference strain (*S. aureus* ATCC 6538) and a clinical strain isolated from a diabetic foot infection. On a previous study^9^ the virulence determinants of this clinical isolate were investigated, revealing the presence of genes involved in biofilm formation and development such as intracellular adhesin genes *icaA* and *icaD*, quorum sensing gene *agrI*, coagulase gene *coa*, protein A gene *spa*, and clumping factor *clfa*.

**Figure 2:**
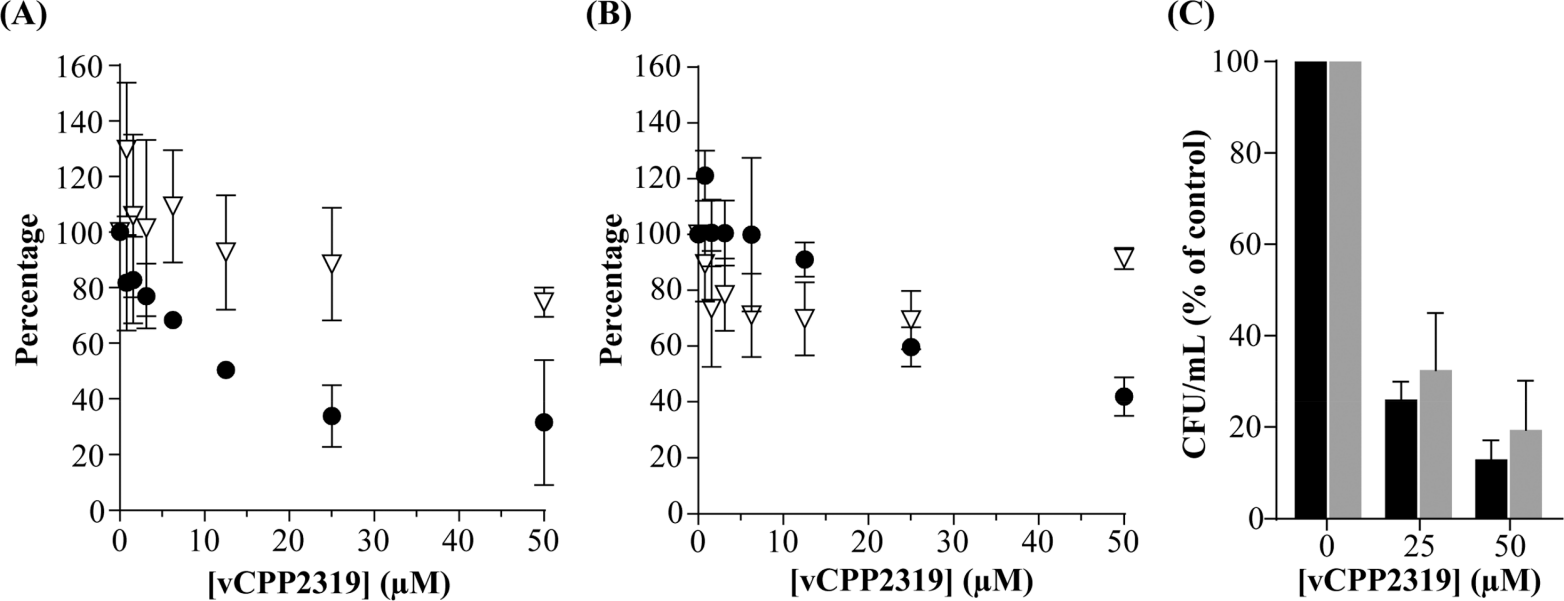
vCPP2319 activity against preformed *S. aureus* biofilms. Effect of vCPP2319 on biofilms produced by *S. aureus* ATCC 6538 (A) and by the *S. aureus* clinical isolate (B). Biofilm-embedded cell metabolic activity was evaluated using the resazurin reduction fluorometric kinetic assay after peptide treatment for 4 h (●). The biofilm biomass was evaluated by using the crystal violet binding assay after peptide treatment for 4 h (▽). Percentages were determined relative to the control (untreated biofilm). (C) Bacterial viability of *S. aureus* ATCC 6538 (black column) and *S. aureus* clinical isolate (gray column) biofilms, untreated and vCPP2319-treated at 25 or 50 μM, for 4 h, was evaluated using a colony count assay. Percentages were determined relative to the control (untreated biofilm).

The activity of vCPP2319 was first evaluated against preformed biofilms formed by *S. aureus* ATCC 6538. Treatment with increasing concentrations of peptide for 4 h (Figure 2A) and 24 h (Figure S3) caused a significant reduction in the metabolic activity of biofilm-embedded cells, in a dose-dependent manner, and irrespective of incubation time. In contrast, when the peptide was used to treat preformed biofilms produced by the clinical isolate for 4 h, only at 25 and 50 μM was the metabolic activity significantly reduced (Figure 2B). The different efficacy of vCPP1319 against each bacterial strain is likely due to the structure of the respective biofilms modulating the peptide accessibility to biofilm-embedded cells, as the effect of vCPP2319 on the planktonic form is similar for both strains (MIC = 3.13 μM). To test this hypothesis, the cell density and EPS matrix of biofilms from both strains were characterized by using confocal microscopy with a combination of two dyes, SYTO 9 and WGA Alexa Fluor 633. SYTO 9 translocates and binds to the intracellular DNA (iDNA) of bacteria with intact and damaged membranes, staining all bacteria green.^32^ For its part, WGA Alexa Fluor 633 binds sialic acid and *N*-acetylglucosamine residues^33^ on the *S. aureus* EPS, staining the biofilm matrix red. As shown in Figure S4, although both biofilms present a dense bacterial cell population, biofilms produced by the *S. aureus* clinical isolate have a denser matrix at both the outer and inner layers, whereas in the reference strain biofilms the matrix localizes mainly at the surface. In a denser matrix, electrostatic binding of cationic vCPP2319 to anionic matrix components will be more extensive, hence increase the amount of peptide needed to reach and act on biofilm cells.

Importantly, for biofilms formed by both strains, colony count assays (Figure 2C) demonstrated that vCPP2319 has a direct effect on the viability of biofilm-embedded cells. Increasing peptide concentration decreased bacterial viability, an effect that, in line with the results above, seems to be more pronounced on the *S. aureus* ATCC 6538 biofilms compared to the clinical isolate biofilms.

Regarding the effect on biomass, for ATCC 6538 biofilms, no significant changes were observed after treatment with vCPP2319 for either 4 or 24 h (Figure 2A and Figure S3). As cells usually account for less than 10% of biofilm biomass, while the EPS matrix can represent over 90% of the dry mass,^3^ these results demonstrate that vCPP2319 does not affect the matrix. However, for clinical isolate biofilms (Figure 2B), vCPP2319 induced a decrease in biomass, suggesting an effect on the matrix. Although uncommon, some studies of AMP effects on biofilm extracellular matrix have been reported. For instance, the AMP piscidin-3 cleaves the eDNA of 24 h-preformed *P. aeruginosa* PA01 biofilms by coordinating with Cu^2+^ through its N-terminus.^34^ For vCPP2319, the biofilm biomass reduction observed was approximately 30%; thus, the peptide impacts the matrix, albeit moderately when compared to its effect on viability of the bacterial cells. Altogether, data show that vCPP2319 acts on *S. aureus* biofilms mainly by targeting bacterial cells, with a limited effect on the EPS matrix.

### vCPP2319 Affects the Morphology and Topography of Preformed *S. aureus* Biofilms

**Figure 3:**
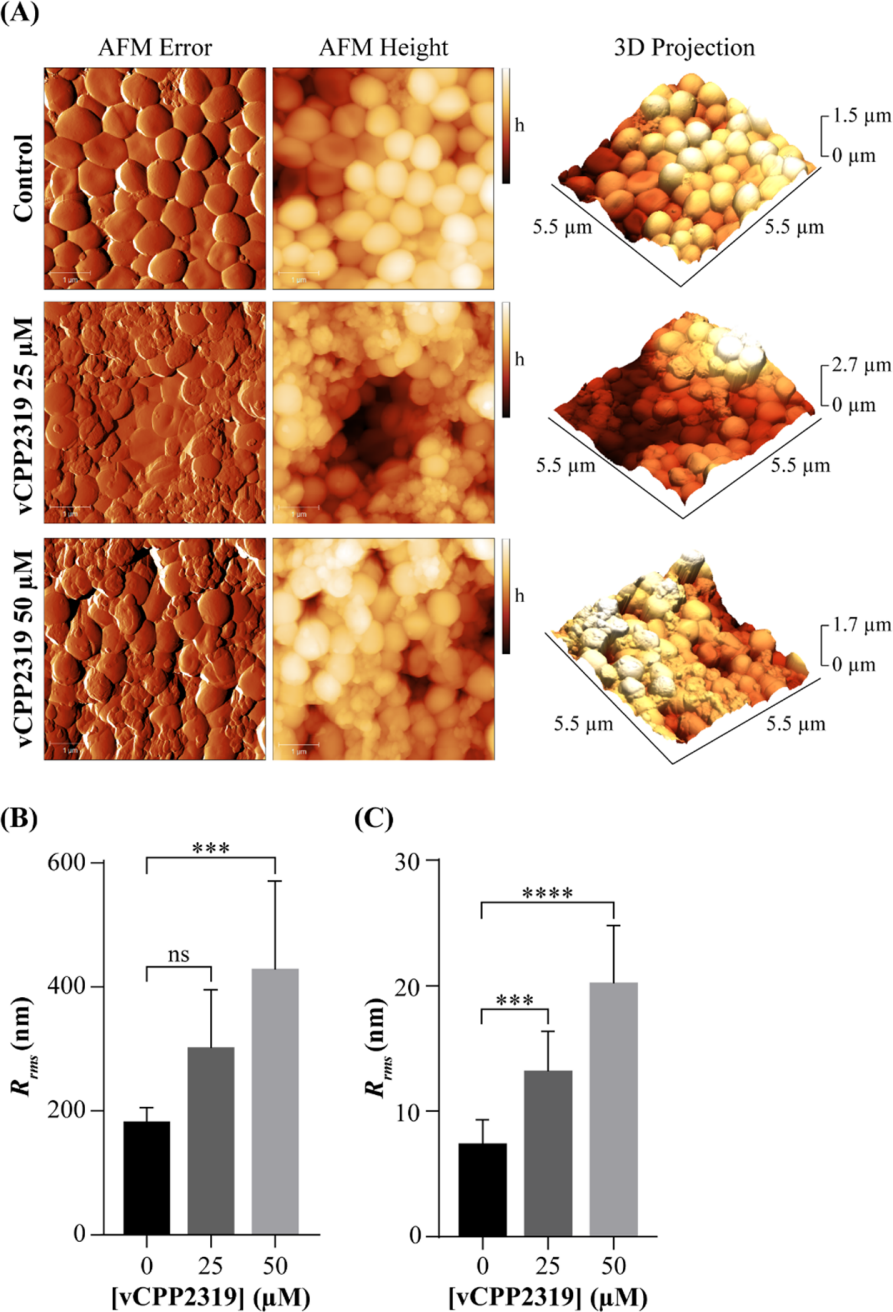
Effect of vCPP2319 on biofilms produced by the *S. aureus* clinical isolate imaged by AFM. (A) Representative AFM error and height images, and respective 3D projections, are presented for untreated biofilms and vCPP2319-treated biofilms at 25 or 50 μM, for 4 h. Total scanning area for each image was 5 × 5 μm. *h*, height. (B) Mean biofilm surface roughness (*R*_*rms*_) of untreated and vCPP2319-treated biofilms. ***p-value ≤0.001. (C) Mean cell surface roughness (*R*_*rms*_) of untreated and vCPP2319-treated biofilms. ***p-value ≤0.001; ****p-value ≤0.0001.

AFM, a technique that allows to evaluate the morphology and topography of biofilms and biofilm-embedded bacterial cells,^35,36^ was used to image the direct effect of vCPP2319 on 24 h-preformed biofilms formed by the *S. aureus* clinical isolate. For untreated biofilms (Figure 3A, top panel), both the representative AFM error and height images and the correspondent 3D projections revealed a dense and uniform layer of cells presenting the characteristic staphylococcal round shape and smooth surface. After treatment with 25 μM vCPP2319 (Figure 3A, middle panel) or 50 μM (Figure 3A, bottom panel) for 4 h, significant changes in the morphology of the biofilms were observed, evidencing a more irregular surface, an effect that was more pronounced at the higher peptide concentration tested. In agreement, quantification of the biofilm topography, through determination of surface roughness (*R*_*rms*_, Figure 3B), showed an increase from 183 ± 23 nm for untreated biofilms to 303 ± 93 and 429 ± 142 nm after treatment with vCPP2319 at 25 and 50 μM, respectively.

The results also showed that the peptide has a direct effect on the microstructure of the bacterial cells, which lost their smooth surface and became more wrinkled. In line with this observation, the *R*_*rms*_ value (Figure 3C) for untreated biofilm-embedded bacterial cells was 7.4 ± 1.9 nm, increasing to 13 ± 3 and 20 ± 5 nm after treatment with vCPP2319 at 25 and 50 μM, respectively. As proposed in another AFM study,^37^ such results might correlate with an increase in the bacterial membrane permeability caused by the peptide.

### vCPP2319 Permeates the Membrane of Biofilm-Embedded Bacterial Cells

**Figure 4:**
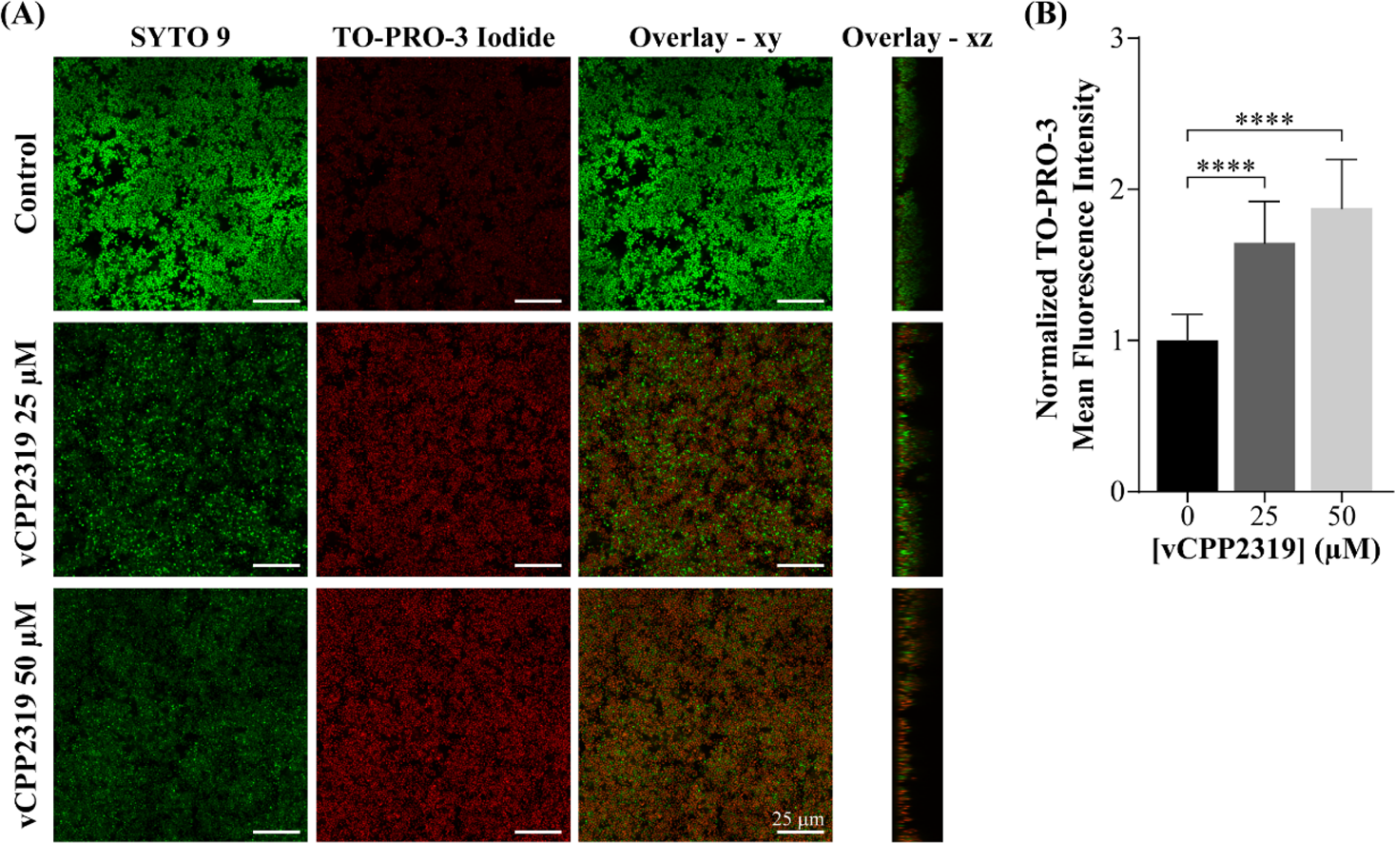
Effect of vCPP2319 on the membrane integrity of *S. aureus* clinical isolate biofilm-embedded bacteria imaged by CLSM. (A) Representative CLSM images corresponding to untreated and vCPP2319-treated biofilms at 25 or 50 μM, for 4 h. Biofilms were stained with the nucleic acid-binding dyes SYTO 9 (green) and TO-PRO-3 iodide (red). The overlay between the green and red channels for the *xy* and *xz* orthogonal plane images are presented. The *xy* plane images were taken at an inner layer of the biofilms (*z* = 2 μm). (B) Normalized TO-PRO-3 iodide mean fluorescence intensity measured for untreated and vCPP2319-treated biofilms. TO-PRO-3 iodide mean fluorescence intensity was calculated from the *xy* plane images (*z* = 2 μm) using Fiji’s incorporated plug-ins. ****p-value ≤0.0001.

To further investigate the ability of vCPP2319 to directly induce membrane permeation of biofilm-embedded bacterial cells, a live/dead CLSM-based assay was performed. Twenty-four hour-preformed *S. aureus* biofilms, untreated or treated with vCPP2319 for 4 h, were sequentially stained with the nucleic acid-binding dyes SYTO 9 and TO-PRO-3 iodide. SYTO 9 translocates and binds iDNA of all bacteria, intact membranes or not, staining bacterial cells green, while TO-PRO-3 iodide only binds the iDNA of bacteria with damaged membranes, staining bacteria red.^38,39^ Representative CLSM images of an inner layer of biofilms (*z* = 2 μm) are shown in Figure 4A. While bacterial cells of untreated biofilms are stained green (Figure 4A, top panel), treatment with 25 and 50 μM vCPP2319 (Figure 4A, middle and bottom panels) resulted in an increase of red-stained bacteria, with a concomitant decrease of green-stained ones (left panels, SYTO 9 and TO-PRO-3 iodide *xy* plane images). This indicates that the peptide damages the membrane of bacteria, enabling TO-PRO-3 iodide to displace, at least partially, SYTO 9 bound to iDNA, which results in bacteria stained orange in the *xy* and *xz* overlay images.

In sum, quantitative analysis of the TO-PRO-3 iodide fluorescence signal at an inner (*z* = 2 μm) biofilm layer (Figure 4B), clearly shows the concentration-dependent ability of vCPP2319 to kill biofilm-embedded cells by permeabilization of their membranes.

### Combined Use of vCPP2319 with α-Amylase

Results so far show that vCPP2319 acts on biofilms mainly by targeting and killing embedded cells. However, even when at 50 μM peptide, about 19 ± 10.8% bacteria survived the treatment (Figure 2C) after 4 h. This might be due to peptide interaction with matrix components, which slows down diffusion and/or the amount of peptide available to target cells at inner layers, as shown for other peptides.^24,40^ To test this hypothesis, 24 h-preformed *S. aureus* clinical isolate biofilms were sequentially treated with two doses of vCPP2319. As shown in Figure S5, after a first treatment with 25 μM vCPP2319 for 4 h, the percentage of viable cells decreased to 44.7 ± 4.3%, followed by a decrease to 27.6 ± 13.6% or 9.4 ± 4.7%, after a second treatment at 25 or 50 μM, respectively. These results confirm a correlation between peptide availability and bacterial killing and suggest that bacteria intrinsically resistant to vCPP2319 are not present in the biofilm.

In view of the above, we further investigated if the combined use with a matrix-degrading agent could facilitate peptide access to bacterial cells. This approach is in line with previous reports where joint use of molecules targeting different biofilm components is shown advantageous over single antibiofilm agents.^41^

As polysaccharides are a major component of the EPS matrix of most bacterial biofilms, we selected the glycoside hydrolase α-amylase from *Bacillus* sp. (EC 3.2.1.1), an enzyme with no hemolytic activity up to 0.5 mg/mL. As shown in Figure S6, α-amylase had a significant concentration-dependent effect on the total biomass of 24 h-preformed clinical isolate biofilms, with ca. 80% reduction at 0.5 mg/mL, the highest concentration tested, confirming its ability to degrade the biofilm matrix. Since biofilm mass reached a plateau, the remaining mass must be assigned to nonsaccharide EPS components, namely eDNA and proteins, and to biofilm-embedded cells. From this dose–response assay, a concentration of 0.13 mg/mL was chosen for further assays as the highest not affecting metabolic activity of embedded cells (results not shown), thus only targeting the matrix.

**Figure 5:**
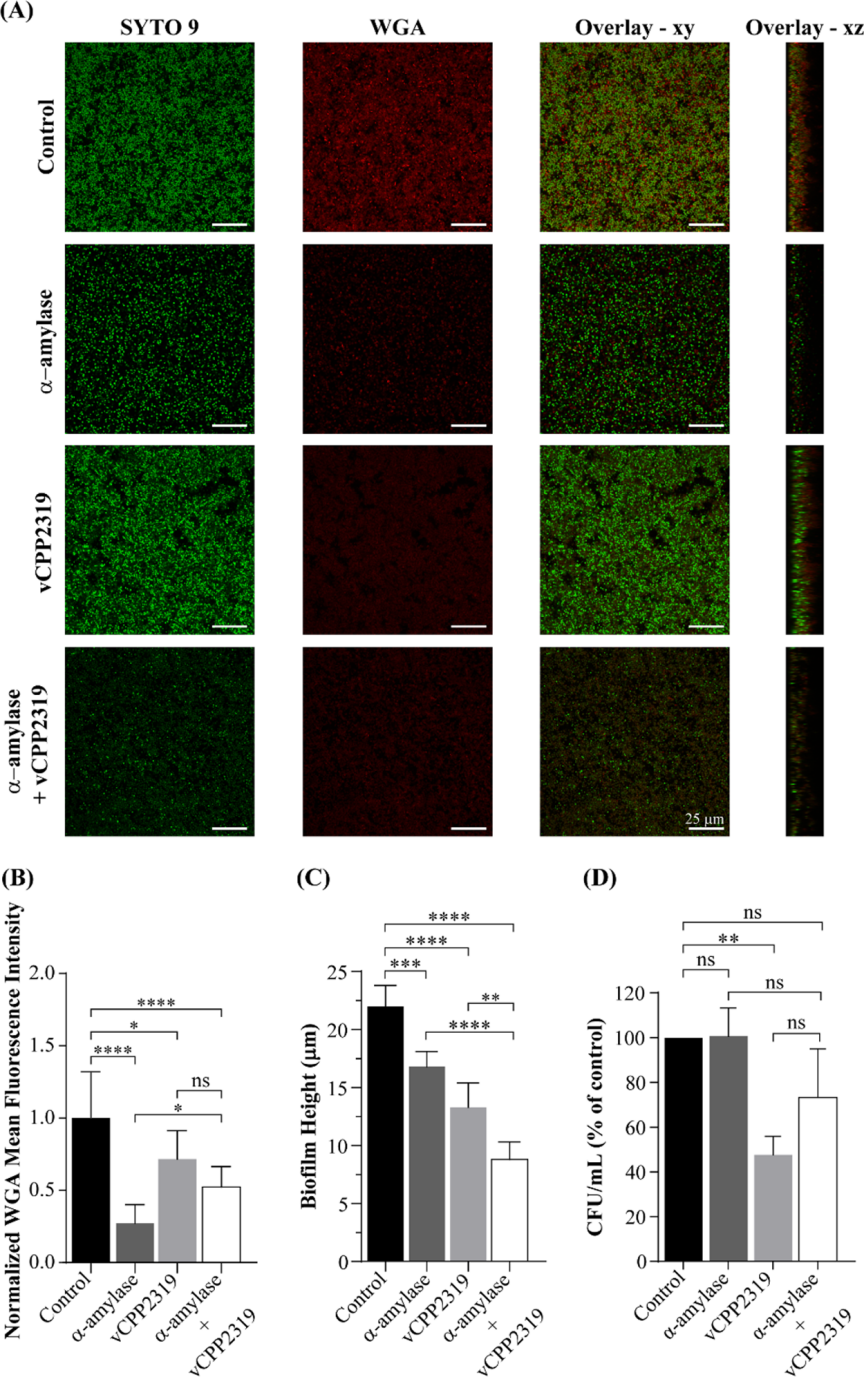
Effect of sequential treatment with α-amylase and vCPP2319 on *S. aureus* clinical isolate biofilms imaged by CLSM. (A) Representative CLSM images corresponding to untreated biofilms, biofilms treated with 0.13 mg/mL α-amylase, 25 μM vCPP2319-treated biofilms, and biofilms treated sequentially with α-amylase and vCPP2319 at the same concentrations. Biofilms were stained with nucleic acid stain SYTO 9 (green) and with the lectin WGA Alexa Fluor 633 (red). The overlay between the green and red channels for the *xy* and *xz* orthogonal plane images are presented. The *xy* plane images were taken at an inner layer of the biofilms (*z* = 2 μm). (B) Normalized WGA mean fluorescence intensity was measured for untreated and treated biofilms. WGA mean fluorescence intensity was calculated from the *xy* plane images (*z* = 2 μm) using Fiji’s incorporated plug-ins. Supplemental Videos compiling sequential *xy*-axis micrographs, taken for untreated and treated biofilms, are shown in the Supporting Information (Videos S1–S4). (C) Biofilm height of untreated and treated biofilms. Biofilm height was determined from the *xz* orthogonal images by using ZEN lite software (Carl Zeiss MicroImaging). (D) Combined effect of vCPP2319 with α-amylase on biofilm-embedded bacteria viability. Bacterial viability was evaluated using a colony count assay. Percentages were determined relative to the control (untreated biofilm). ns = nonsignificant; *p-value ≤0.05; **p-value ≤0.01; ***p-value ≤0.001; ****p-value ≤0.0001.

To further elucidate the effect of α-amylase on the structure of 24 h-preformed clinical isolate biofilms, we used a CLSM-based assay and stained the biofilms with SYTO 9 and WGA Alexa Fluor 633. Representative CLSM images of untreated biofilms (Figure 5A, top panel) show a dense population of green-stained cells surrounded by a red-stained polysaccharide-rich EPS matrix. When biofilms were incubated with 0.13 mg/mL α-amylase, there was a clear decrease in red staining (Figure 5A, second panel). There is also a decrease in the intensity of WGA fluorescence of the *z* = 2 μm plane, Figure 5B, indicative of a decreased density of biofilm polysaccharide. This is consistent with the ability of the enzyme to degrade the matrix, as found with the crystal violet assay (Figure S6). The peptide vCPP2319 (25 μM) has a similar effect but not as pronounced, and the sequential addition of α-amylase and vCPP2319 has an intermediate effect (Figure 5B). Concomitantly, as shown by Video S2, bacterial mobility increases, indicative of a “looser” (less dense) matrix; a similar effect is not detected with the peptide alone (Video S3). Intriguingly, biofilm height is more affected by the peptide than by α-amylase; the combined effect is stronger (Figure 5C). Calculation of relative polysaccharide density caused by the enzyme or the peptide relative to control can be performed directly from the WGA fluorescence values at *z* = 2 μm (eq 4) or the integrated fluorescence intensities of WGA over *zz* divided by biofilm height (eq 6). The severe effect of α-amylase compared to peptide is confirmed (Table 1). Peptide addition after digestion with the enzyme causes compaction of the biofilm to levels observed with the peptide alone.

**Table 1:** Fold Variation Relative to Control (Untreated Biofilm) of the Biofilm Density, ρ, and Mass, *m*, after the Addition of α-Amylase, vCPP2319, or the Sequential Addition of Both

	ρ_*i*_/ρ_control_			*m*_*i*_/*m*_control_	
	(polysaccharides)		(global)	(polysaccharides)	(global)
*i*	eq 4	eq 6	eq 8	eq 5	eq 7
α-amylase	0.3	0.4	0.4	0.3	0.3
vCPP2319	0.7	0.8	1.2	0.5	0.7
α-amylase + vCPP2319	0.5	0.5	0.8	0.2	ND^a^

a ND = not determined.

Taking into account the integrated WGA fluorescence emission over the total biofilm, one can directly calculate the total mass loss relative to the control (eq 5) caused by the enzyme, the peptide, or both (Table 1). The enzyme caused a high polysaccharide loss, while the peptide had a milder but still pronounced effect.

The total (instead of polysaccharide-only) mass and density variations of the biofilm can be calculated from the results of the crystal violet staining assay (Figure 2B and Figure S6) using eqs 7 and 8. Results are in line with those obtained for polysaccharides only (WGA staining), but the impact of α-amylase is smaller (Table 1), as expected because the enzyme does not affect nucleic acids and proteins in the biofilm matrix.

The synergy between α-amylase and vCPP2319 in reducing the biofilm height is worth noting. For two independent events, the probability of a simultaneous occurrence is the product of the probabilities of the individual events. Applied to the data in Figure 5C this would result in a height reduction to 46% relative to control. Instead, a reduction to 36% is observed, suggesting that the peptide can shrink the biofilm to a slightly larger extent when polysaccharides are previously removed. This effect may be related to direct exposure of nucleic acids to the polycationic peptide with ensuing cross-bridging of the matrix. Importantly, previous digestion of the biofilm by α-amylase does not improve the antibacterial activity of the peptide as evaluated from a colony count assay (Figure 5D), which suggests that the limited activity of the peptide is determined by its interaction with the anionic nucleic acids of the matrix, irrespective of the presence of the polysaccharides. In line with this hypothesis, when using nucleic acids-digesting enzymes combined with other AMPs, synergy in antimicrobial activity is observed^42,43^

## Conclusions

Biofilm-related infections are extremely challenging to treat, making the development of new and effective therapeutic strategies urgently needed. Here, we showed that the viral-derived peptide vCPP2319, known for its activity against bacteria in planktonic form, is also able to act against biofilms by killing bacterial cells, highlighting its potential as a dual-action AMP. Using quantitative imaging methods, we also demonstrated that the peptide acts by targeting and disrupting the membrane of biofilm-embedded cells. Importantly, we showed that vCPP2319 has remarkable stability in human serum as well as low hemolytic and cytotoxic activities. Moreover, sequential treatment of *S. aureus* biofilms with vCPP2319 in combination with α-amylase, an EPS-degrading enzyme, revealed the impact of both agents on global structure, i.e., compactness, of the biofilm. Taking together antimicrobial activity and AFM results, the following conclusions can be advanced:

1. Polysaccharides are a relatively loose component of the 24-h biofilm: it is not visualized in AFM imaging; its mass is partially reduced by the vCPP2319, and greatly reduced by α-amylase.
2. As a result of α-amylase action, polysaccharide density in the biofilm is severely reduced as evidenced by bacterial motion and data in Table 1. The same happens with the peptide albeit not as severely.
3. α-Amylase action also impacts on biofilm density as a whole (global biofilm mass considered) by decreasing it. The peptide has a different effect, as it densifies the biofilm: the reduction in height balances the reduction in mass.
4. When the peptide acts after biofilm digestion by α-amylase, compactness is partially restored despite polysaccharide loss. The cationic peptide probably crossbridges anionic nucleic acids in the matrix, increasing its density.
5. The compaction effect of the peptide compensates the digesting effect of α-amylase abrogating synergy in antimicrobial activity (Figure 5D). This suggests that combining polysaccharide-digesting agents with antibiotic polyelectrolytes such as AMPs is not a suitable strategy to treat bacterial biofilms. In contrast, a crossbridging effect is not observed when using nucleic acids-digesting enzymes combined with AMPs, thus ensuring synergy.^42,43^

## Methods

### Peptide Synthesis

vCPP2319, WRRRYRRWRRRRRWRRRPRR-amide,^27,28^ was synthesized by Bachem AG (Bubendorf, Switzerland) with a purity of >95%. The peptide has an amidated C-terminus and a free amine N-terminus. To prepare stock solutions, lyophilized peptide was weighed out on a high precision analytical microbalance, dissolved in sterile Milli-Q water, and stored at −20 °C.

### Hemolytic Activity

Fresh human blood was obtained from healthy donors after written informed consent. To isolate human red blood cells (hRBCs), samples were centrifuged at 1000*g* for 10 min at 4 °C, washed three times, and resuspended in sterile phosphate buffered saline (PBS) (pH 7.4) to a final concentration of 0.25% (v/v). hRBCs suspensions were incubated with vCPP2319, ranging from 0.05 to 50 μM, for 4 or 24 h, with α-amylase from *Bacillus* sp. (EC 3.2.1.1; ∼380 U/mg; Cat no. 10069 from Sigma-Aldrich), ranging from 0.0078 to 0.5 mg/mL, for 1 h, or with the hemolytic peptide melittin (positive control), ranging from 0.05 to 50 μM, for 1 h at 37 °C, with gentle swirling, in a sterile 96-well microtiter round-bottomed polypropylene plate (Corning, NY, USA). After incubation, the plate was centrifuged at 1000*g* for 5 min at 4 °C, and supernatants were transferred to a sterile 96-well microtiter clear flat-bottomed polystyrene plate (Corning). Hemoglobin release from lysed cells was quantified by measuring the absorbance at 415 nm in an infinite M200 microplate reader (Tecan, Männedorf, Switzerland). Samples incubated with PBS were used as negative control, and samples incubated with Triton X-100 at 1% (v/v in H_2_O) were used as a control for membrane-disruption.^44^ Hemolytic activity (%) was determined using the following equation:
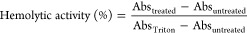
1where Abs_treated_ is the absorbance of peptide-treated cells, Abs_untreated_ is the absorbance of samples incubated with PBS, and Abs_Triton_ is the absorbance of samples incubated with Triton X-100. HC_50_ values were inferred from sigmoidal dose–response (variable slope) curves, using GraphPad Prism 7.0 software (GraphPad Software, San Diego, CA, USA). Experiments were performed on different days using different human blood samples.

### Cell Culture and Cytotoxicity

Human dermal microvascular endothelium cells (HMEC-1, ATCC CRL-3243) (ATCC, Manassas, VA, USA) were cultured as a monolayer using MCDB-131 medium (without l-glutamine) with phenol red (Gibco, Thermo-Fisher, Waltham, MA, USA). The medium was supplemented with 10% (v/v) fetal bovine serum (FBS), 1% (v/v) penicillin/streptomycin (Gibco, Thermo-Fisher), 10 ng/mL epidermal growth factor (EGF), 1 μg/mL hydrocortisone (Sigma-Aldrich, St. Louis, MO, USA), and 10 mM glutamine (ATCC), according to the manufacturer’s instructions. Cells were grown in a humidified atmosphere of 5% CO_2_ at 37 °C. The cytotoxicity of vCPP2319 toward HMEC-1 cells was determined using CellTiter-Blue Cell Viability Assay (Promega, USA), according to the manufacturer’s instructions. HMEC-1 cells were seeded into a sterile 96-well clear flat-bottomed polystyrene plate (Corning) at 2.5 × 10^4^ cells/well and incubated for 24 h at 37 °C. After medium removal, cells were washed twice with PBS. Then, 100 μL of previously diluted vCPP2319 (0.01–50 μM) in MCDB-131 medium was added to the wells. After 24 h of incubation with vCPP2319, cells were washed twice with PBS and 20 μL of CellTiter-Blue reagent in 100 μL of MCDB-131 medium was added to each well and incubated for 3 h in a humidified atmosphere of 5% CO_2_ at 37 °C. The fluorescence intensity was measured with excitation and emission at 560 and 590 nm, respectively, using the infinite M200 microplate reader (Tecan). Medium and 1% (v/v) Triton X-100-containing medium were used as positive control and negative control, respectively. HMEC-1 cells metabolic activity (%) was determined using the following equation:
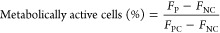
2where *F*_P_ is the fluorescence intensity of peptide-treated cells, *F*_NC_ is the fluorescence intensity of negative controls, and *F*_PC_ is the fluorescence intensity of positive controls. Experiments were performed on different days using independently grown cell cultures.

### Stability in Human Serum

vCPP2319 at final concentrations of 50, 100, 200, and 500 μM was incubated with 25% (v/v in H_2_O) human serum (Sigma-Aldrich) at 37 °C, with gentle swirling. At different time points (0, 1, 5, 10, 30, 60 120, 360, and 1440 min), 120 μL aliquots were taken and treated with 20 μL of 5% perchloric acid (v/v in H_2_O) for 30 min at 4 °C, to stop the proteolytic reaction. To determine the maximum peptide intensity (100% intact peptide; *t*_0_), serum proteins were first precipitated, followed by the addition of the peptide. Controls of serum without peptide and peptide not incubated in serum were also included in the assay. Samples were next centrifuged at 13 000*g* for 10 min to remove serum proteins. The supernatants were analyzed by reversed-phase HPLC (RP-HPLC) and LC–mass spectrometry (LC-MS). The percentage of intact peptide was calculated by RP-HPLC peak integration, expressed as percent of the amount at *t*_0_, and data were fitted to a monoexponential decay model using GraphPad Prism 7.0 to estimate the peptide half-life (*t*_1/2_). Analytical RP-HPLC was performed on a LC-20AD instrument (Shimadzu, Kyoto, Japan) equipped with a Luna C18 column (4.6 × 50 mm, 3 μm; Phenomenex, Torrance, CA, USA) using linear gradients of solvent B (0.036% (v/v) trifluoroacetic acid (TFA) in acetonitrile (ACN)) into A (0.045% (v/v) TFA in H_2_O) over 15 min, at a flow rate of 1 mL/min and UV detection at 220 nm. MS analysis was performed on an LC-MS 2010EV instrument (Shimadzu) fitted with an XBridge C18 column (4.6 mm × 150 mm, 3.5 μm, Waters, Cerdanyola del Valles, Spain), eluting with linear gradients of F (0.08% (v/v) formic acid [FA] in ACN) into E (0.1% (v/v) FA in H_2_O) over 15 min at 1 mL/min flow rate.^45^

### Dynamic Light Scattering

DLS experiments were carried out on a Zetasizer Nano ZS instrument from Malvern (Worcestershire, UK). vCPP2319, in PBS, was preincubated for 4 h, at 25 °C, at different concentrations (50, 100, 200, and 500 μM). The Z-average hydrodynamic diameter was measured using DLS in particle size analysis mode. For each experiment, a single measurement, corresponding to an autocorrelation curve averaged from a minimum of 20 runs, was performed. All measurements were performed at 25 °C. Data were obtained from two independent experiments.

### Bacterial Strains and Growth Conditions

*S. aureus* ATCC 6538 was purchased from ATCC. *S. aureus* clinical strain was previously isolated from patients with infected foot ulcers, genotyped and screened for virulence and antimicrobial resistance traits.^9^ Bacteria in the planktonic form were grown in Mueller Hinton Broth (MHB) (BD, Franklin Lakes, NJ, USA), for 18 h at 37 °C. *S. aureus* bacterial biofilms were grown in tryptic soy broth (TSB) (BD) containing 0.25% (w/v) glucose (TSBG) (Sigma-Aldrich) for 24 h at 37 °C, to allow biofilm formation.

### Activity against Planktonic Bacteria

The ability of vCPP2319 to inhibit bacterial growth was evaluated by determining the minimal inhibitory concentration (MIC) using a standard broth microdilution procedure,^46,47^ as previously described.^28^ Briefly, *S. aureus* suspensions were prepared in MHB to a final concentration of 1 × 10^6^ CFU/mL and incubated for 18 h at 37 °C in a sterile 96-well microtiter round-bottomed polypropylene plate (Corning), containing 2-fold dilutions of vCPP2319. Final bacterial concentration was 5 × 10^5^ CFU/mL whereas peptide concentration ranged from 0.78 to 100 μM. The MIC was defined as the lowest peptide concentration required to inhibited visible bacterial growth.

### Activity against Bacterial Biofilms

The effect of vCPP2319 on 24 h-preformed *S. aureus* biofilms was evaluated using three distinct methods, as previously described:^24^ the metabolic activity and viability of biofilm-embedded cells was determined using a resazurin reduction fluorometric assay and a colony count assay, respectively. The total biofilm biomass was quantified by using a crystal violet assay.

#### Resazurin Reduction Fluorometric Assay

Resazurin, the active compound in alamarBlue reagent (Invitrogen), is a blue dye that can be reduced to a pink fluorescent intermediate, resorufin, as a result of cells metabolic activity.^48^*S. aureus* suspensions were prepared to a final concentration of 1 × 10^6^ CFU/mL and incubated in a sterile 96-well microtiter black flat-bottomed polystyrene plate (Corning) for 24 h at 37 °C. Nonadherent bacteria were removed after a washing step with MHB, and preformed biofilms were incubated in the absence or presence of 2-fold serial dilutions of vCPP2319, ranging from 0.78 to 50 μM, for 4 or 24 h at 37 °C. Untreated biofilms were used as a control. After washing the biofilms twice with MHB, alamarBlue reagent was added at a final concentration 10% (v/v in MHB) to each sample and its reduction was monitored by measuring the fluorescence intensity (excitation and emission wavelengths were 530 and 590 nm, respectively) of the samples every 5 min during 2 h at 37 °C, in an infinite M200 microplate reader (Tecan). Fully reduced resazurin in MHB, obtained after autoclaving the sample for 15 min, was used as positive control. The percentage of resazurin reduction was determined relatively to the control, after blank (10% (v/v) alamarBlue reagent in MHB) correction.

#### Colony Count Assay

*S. aureus* suspensions at 1 × 10^6^ CFU/mL were incubated in a sterile 96-well microtiter clear flat-bottomed polystyrene plate (Corning) for 24 h at 37 °C. Nonadherent bacteria were removed after a washing step with MHB, and preformed biofilms were incubated in the absence or presence of vCPP2319 at 25 or 50 μM, for 4 h at 37 °C. Untreated biofilms were used as a control. To evaluate the effect of vCPP2319 redosing on biofilm-embedded bacteria, 24 h-preformed biofilms were incubated in the absence or presence of vCPP2319 at 25 μM, for 4 h at 37 °C. After a washing step with MHB, biofilms were further incubated in the absence or presence of an additional dose of vCPP2319 at 25 or 50 μM, for 4 h at 37 °C. In both experimental settings, after two washing steps with PBS, the biofilms were resuspended in PBS, followed by cell scrapping with a pipet tip as described elsewhere.^49,50^ To evaluate the combined effect of vCPP2319 with α-amylase from *Bacillus* sp. (EC 3.2.1.1) on biofilm-embedded bacteria, 24 h-preformed biofilms were incubated with 0.13 mg/mL α-amylase for 4 h, followed by the addition of 25 μM vCPP2319, without washing, for another 4 h at 37 °C. Controls of untreated biofilms and biofilms treated with α-amylase or vCPP2319 individually were prepared on the same conditions as the ones used for the combination of the molecules to have the same total time of incubation. After incubation, the biofilms were removed by cell scrapping with a pipet tip as described elsewhere.^49,50^ In all experimental settings, aliquots were vortexed at high speed, serially diluted in PBS and plated on nutrient-rich trypcase soy agar (TSA) plates (bioMérieux, Marcy l’Etoile, France). After incubation for 24 h at 37 °C, bacterial colonies were counted, and viable bacteria (in CFU/mL) were reported as percentage of the control.

#### Crystal Violet Binding Assay

Crystal violet is a basic dye that binds to negatively charged molecules, such as those in biofilms EPS matrix and on bacterial membrane surface.^51^*S. aureus* suspensions at 1 × 10^6^ CFU/mL were incubated in a 96-well microtiter clear flat-bottomed polystyrene plate (Corning) for 24 h at 37 °C. After a washing step with MHB to remove nonadherent bacteria, preformed biofilms were incubated in the absence or presence of 2-fold serial dilutions of vCPP2319, ranging from 0.78 to 50 μM, for 4 or 24 h at 37 °C, or with α-amylase from *Bacillus* sp. (EC 3.2.1.1), ranging from 0.0078 to 0.5 mg/mL, for 4 h at 37 °C. Untreated biofilms were used as a control. Biofilms were washed twice with MHB and incubated with 0.25% (v/v in sterile Milli-Q water) crystal violet (Sigma-Aldrich) for 30 min at room temperature. After each sample was washed three times with MHB, crystal violet was solubilized with 95% (v/v) ethanol (Carlo Erba Reagents S.A.S., France) by repeated pipetting. Biofilm biomass was quantified by measuring the absorbance at 590 nm of each sample in an infinite M200 microplate reader (Tecan). The percentage of crystal violet staining was determined relative to the control after blank (0.25% (v/v) crystal violet in sterile Milli-Q water) correction.

### Biofilm Imaging Using Atomic Force Microscopy (AFM)

AFM was used to visualize the topography and morphology of *S. aureus* biofilms in the absence and presence of vCPP2319. Bacterial suspensions at 1 × 10^6^ CFU/mL were incubated in a sterile slide with a removable 12 well silicone chamber (Ibidi) for 24 h at 37 °C. After a washing step with MHB, preformed biofilms were incubated in the absence or presence of vCPP2319 at 25 or 50 μM, for 4 h at 37 °C. Untreated and treated biofilms were then washed once with sterile Milli-Q water and incubated with 0.1% (v/v) glutaraldehyde (Sigma-Aldrich) for 4 h at room temperature. Afterward, each sample was washed twice with sterile Milli-Q water and allowed to dry in air for 1 h at room temperature. AFM images were acquired by using a JPK NanoWizard IV (Berlin, Germany) mounted on a Zeiss Axiovert 200 inverted microscope (Oberkochen, Germany). The AFM head was equipped with a 15 μm z-range linearized piezoelectric scanner and an infrared laser. Images were acquired in air and in intermittent contact mode using uncoated silicon ACL cantilevers from AppNano (Mountain View, CA, USA) with typical resonance frequencies of 200–400 kHz and spring constant of 13–77 N/m. Scan speeds ranged between 0.2 and 0.4 Hz and total scan areas of 5 × 5 μm were imaged. Height and error images were recorded. Height images reflect the topography of the sample. Error images are generated by the deflection of the cantilever as it bends while interacting with the sample and are adequate to improve resolution on the edges of objects imaged by AFM. For each experiment, three different areas were imaged. The surface roughness was defined as the root-mean-square roughness (*R*_*rms*_)^52^ and it was obtained from AFM height images using Gwyddion software version 2.56, based on the following equation after compensation for bacterial curvature:
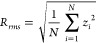
3where *N* is the number of data points and *z*_*i*_ is the height deviation of *i-*th point from a mean line.^53^ For the biofilm surface roughness, *R*_*rms*_ values were calculated from the total imaged area of the biofilm, while for the cell surface roughness, *R*_*rms*_ values were calculated from a line of cells in three different regions of each image of untreated and peptide-treated biofilms. The final *R*_*rms*_ values are the averages of all measurements.

### Biofilm Imaging Using Confocal Laser Scanning Microscopy (CLSM)

To visualize the effect of vCPP2319 on membrane integrity of biofilm-embedded bacteria, an adapted live/dead assay using SYTO 9 and TO-PRO-3 iodide (Invitrogen) was performed, as previously described.^24^*S. aureus* suspensions at 1 × 10^6^ CFU/mL were incubated in a sterile μ-Slide 8-well plate (Ibidi) for 24 h at 37 °C. Preformed biofilms were washed once with PBS and incubated with vCPP2319 at 25 or 50 μM, in PBS, for 4 h at 37 °C. Untreated biofilms were used as a control. After a washing step with PBS, biofilms were sequentially stained with 3 μM SYTO 9 for 30 min and 4 μM TO-PRO-3 iodide for 15 min at room temperature.

The effect of vCPP2319 and/or α-amylase from *Bacillus* sp. (EC 3.2.1.1) on the EPS matrix of *S. aureus* biofilms was evaluated using a CLSM-based assay. Briefly, the bacterial cells and the matrix components of *S. aureus* biofilms were imaged using SYTO 9 and Wheat Germ Agglutinin (WGA) Alexa Fluor 633 Conjugate (Invitrogen, Carlsbad, CA, USA). Preformed biofilms (see above) were washed once with PBS and stained with 3 μM SYTO 9 and 5 μg/mL WGA Alexa Fluor 633, for 15 min at room temperature. After an additional washing step with PBS, biofilms were first incubated with 0.13 mg/mL α-amylase for 4 h, followed by a washing step with PBS, and then further incubated with 25 μM vCPP2319, for another 4 h at 37 °C. Controls of untreated biofilms and biofilms treated with α-amylase or vCPP2319 individually were prepared under the same conditions used in the combined assay to have the same number of washing steps and total time of incubation.

SYTO 9 was excited using the Argon laser (488 nm), and both TO-PRO-3 iodide and WGA Alexa Fluor 633 were excited with the Helium–Neon laser (633 nm), respectively. Image acquisition was performed on a Zeiss LSM 710 confocal laser point-scanning inverted microscope (Carl Zeiss MicroImaging, Oberkochen, Germany) equipped with a Plan-Apochromat DIC 63× oil immersion objective (1.40 numerical aperture). For each experiment, three different areas were imaged. All images were analyzed with the image processor Fiji.^54^ TO-PRO-3 mean fluorescence intensity over the image was quantified from the *xy* plane of an inner layer (*z* = 2 μm, *z* being the distance from the surface of the glass slide) using Fiji’s incorporated plug-ins.^54^ WGA mean fluorescence intensity over the image was quantified from the *xy* plane of an inner layer (*z* = 2 μm, *z* being the distance from the surface of the glass slide) and also from a z-projection of the sum of the z-stack slices, using Fiji’s incorporated plug-ins.^54^ Biofilm height was determined from the *xz* orthogonal images using ZEN lite software (Carl Zeiss MicroImaging).

### Calculation of the Relative Variations in Biofilm Density and Mass

The mean emission fluorescence intensity of WGA integrated over a plane *xy* at a specific depth, *z*, *I*_*f*,WGA,*xy*_, is proportional to the mass of polysaccharides in that plane. Eq 4 allows us to determine the polysaccharide density ratio of the biofilm in condition *i* (presence of α-amylase or peptide, or both) relative to control.
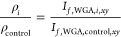
4Integration of fluorescence intensities over *zz* covering the total height (*h*) of the biofilm, *I*_*f*, WGA,*xyz*_, is proportional to the total mass (*m*) of the polysaccharide of the biofilm, and eq 5 can be applied to retrieve the polysaccharide mass ratio of the biofilm in condition *i* relative to control.
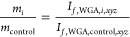
5Alternatively, the polysaccharide density ratio of the biofilm in condition *i* relative to the control can be calculated using eq 6, using *I*_*f*,WGA,*i*,*xyz*_ and *h*_*i*_.
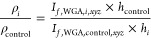
6The global density ratio can be calculated from the crystal violet data, which stain the global mass of the biofilm, using eqs 7 and 8, in which *f*_*i*_ is the fraction of crystal violet absorbance retained in condition *i* relative to control and *V*_*i*_ is the volume of the biofilm in condition *i*. *f*_*i*_ equals the global mass ratio of the biofilm is condition *i* relative to control.
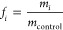
7
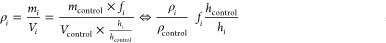
8To apply eq 8 in the presence of both α-amylase and vCPP2319, we assumed the *f*_*i*_ value as the same as that obtained for α-amylase alone.

### Statistical Analysis

Data are described as mean ± standard deviation (SD) of three independent experiments, unless otherwise stated. Statistical significance was assessed by a one-way ANOVA test followed by Tukey’s multiple comparison test and was considered for *p* < 0.05.

## Data Availability

All data supporting the findings of this study is included the article and the online Supporting Information.

## Supporting Information Available

vCPP2319 stability in human serum (Figure S1), vCPP2319 self-assembly (Figure S2), vCPP2319 activity against preformed *S. aureus* biofilms (Figure S3), Characterization of the cellular density and EPS matrix of *S. aureus* biofilms (Figure S4), Effect of vCPP2319 redosing on *S. aureus* biofilms (Figure S5), Antibiofilm properties of α-amylase (Figure S6) (PDF)
*S. aureus* untreated biofilms (Video S1) (MP4)
*S. aureus* biofilms treated with α-amylase (Video S2) (MP4)
*S. aureus* biofilms treated with vCPP2319 (Video S3) (MP4)
*S. aureus* biofilms treated with α-amylase and vCPP2319 added sequentially (Video S4) (MP4)
