# Supplementary material for: Quantitative Imaging of the Action of vCPP2319, an Antimicrobial Peptide from a Viral Scaffold, against Staphylococcus aureus Biofilms of a Clinical Isolate
Source: ACS Infect Dis. 2023 Sep 5;9(10):1889–900. doi: 10.1021/acsinfecdis.3c00195 (PMC10580319; doi:10.1021/acsinfecdis.3c00195)
Supplement: Supplementary file 1 — id3c00195_si_001.pdf [file id3c00195_si_001.pdf]

Supporting Information

Quantitative imaging of the action of vCPP2319, an antimicrobial peptide from a viral scaffold, against *Staphylococcus aureus* biofilms of a clinical isolate

Susana A. Dias[1], Sandra N. Pinto[2], Ana S. Silva-Herdade[1], Marco Cavaco[1], Vera Neves[1], Luís Tavares[3,4], Manuela Oliveira[3,4], David Andreu[5], Ana Coutinho[2,6], Miguel A.R.B. Castanho[1*], Ana Salomé Veiga[1*]

[1]Instituto de Medicina Molecular, Faculdade de Medicina, Universidade de Lisboa, Av. Prof. Egas Moniz 1649-028 Lisboa, Portugal;
[2]iBB-Institute for Bioengineering and Biosciences and Associate Laboratory i4HB - Institute for Health and Bioeconomy at Department of Bioengineering, Instituto Superior Técnico, Universidade de Lisboa, Av. Rovisco Pais 1049-001 Lisboa, Portugal;
[3]CIISA – Centro de Investigação Interdisciplinar em Sanidade Animal, Faculdade de Medicina Veterinária, Universidade de Lisboa, Av. da Universidade Técnica, 1300-477 Lisboa, Portugal;
[4]Laboratório Associado para Ciência Animal e Veterinária (AL4AnimalS), Portugal;
[5]Department of Medicine and Life Sciences, Pompeu Fabra University, Barcelona Biomedical Research Park, Dr. Aiguader 88, 08003 Barcelona, Spain;
[6]Departamento de Química e Bioquímica, Faculdade de Ciências, Universidade de Lisboa, Campo Grande 1749-016 Lisboa, Portugal

***Corresponding authors:**
Miguel A.R.B. Castanho (macastanho@medicina.ulisboa.pt) and Ana Salomé Veiga (aveiga@medicina.ulisboa.pt)

1 - vCPP2319 stability in human serum

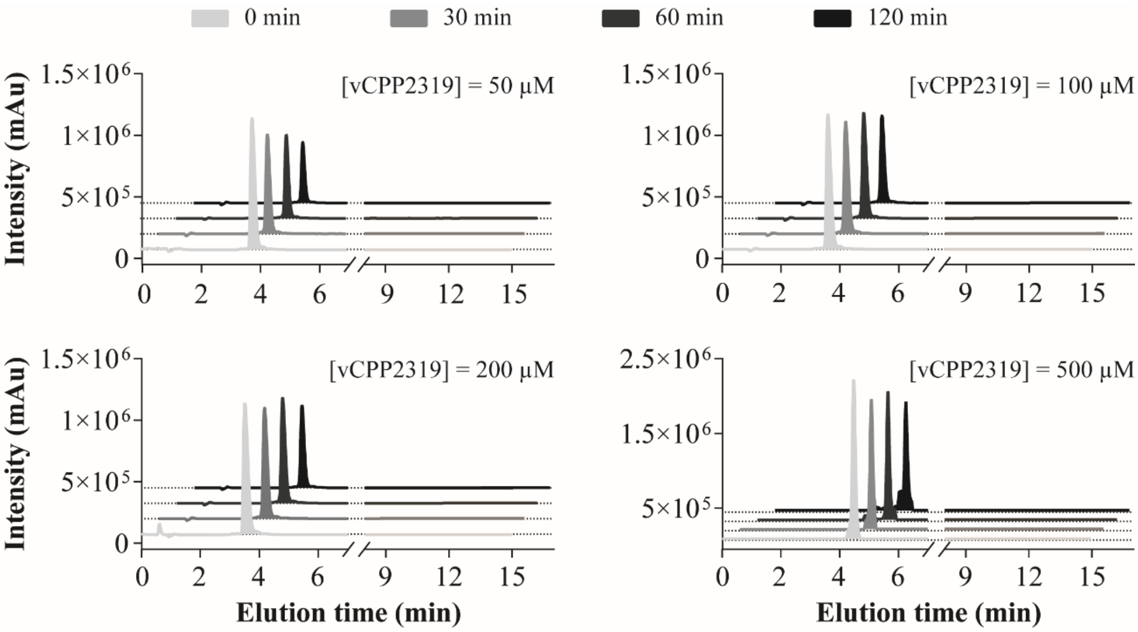

Figure S1 – vCPP2319 stability in human serum. Variable concentrations of the peptide were incubated with 25% (v/v in H_2O) human serum for 0, 30, 60 and 120 min. The intact peptide was separated from degradation products by HPLC using a 0 – 95% ACN gradient over 15 min. Afterwards, the percentage of intact peptide (Figure 1C) was calculated by chromatograms peak integration.

2 - vCPP2319 self-assembly

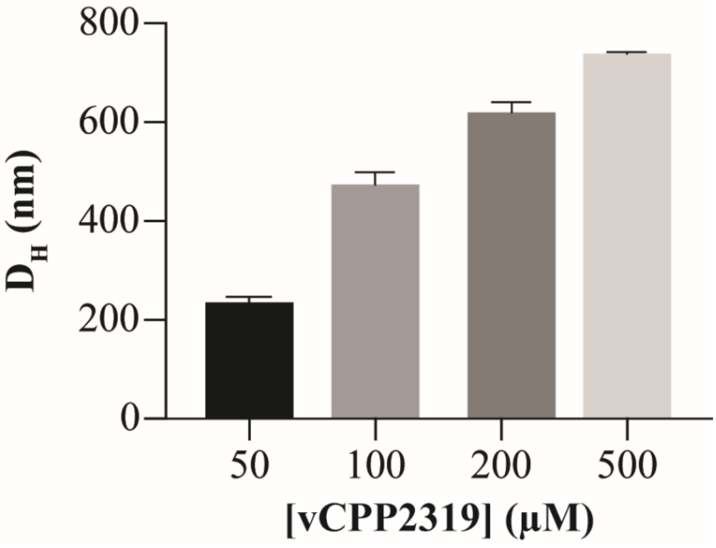

Figure S2 – vCPP2319 self-assembly. Z-average hydrodynamic diameter of the particles formed by different concentrations of vCPP2319 (50, 100, 200 and 500 µM) obtained by dynamic light scattering after 4 h of pre-incubation. Data sets are the average of two independent experiments. Error bars correspond to the standard deviation of the mean.

3 - vCPP2319 activity against preformed *S. aureus* biofilms

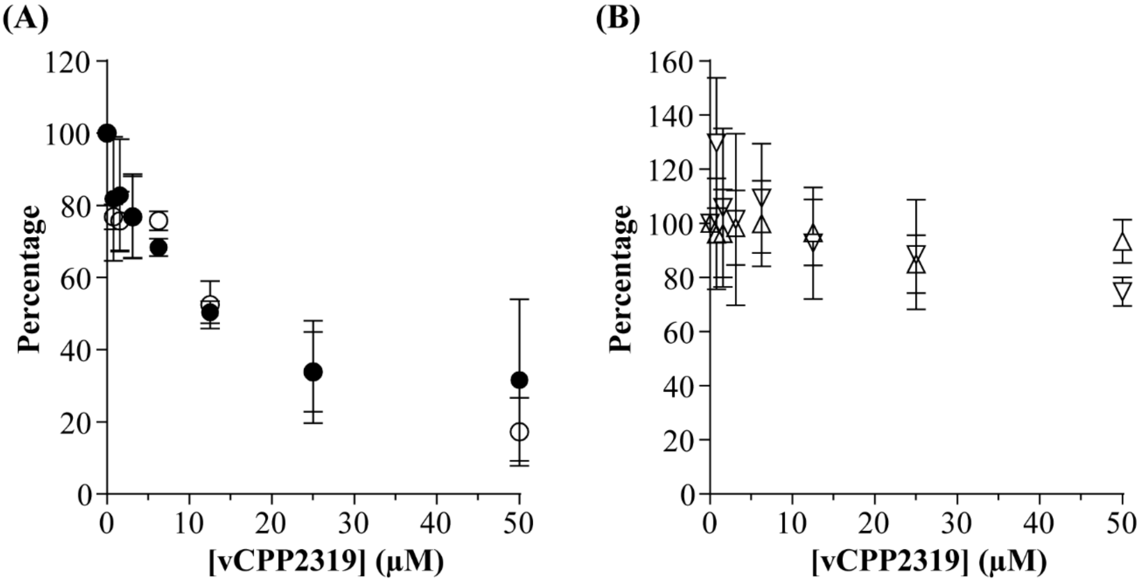

Figure S3 – vCPP2319 activity against preformed *S. aureus* biofilms. Effect of vCPP2319 on biofilms produced by *S. aureus* ATCC 6538. (A) Biofilm-embedded cells metabolic activity was evaluated using the resazurin reduction fluorometric kinetic assay after peptide treatment for 4 h (●) or 24 h (○). (B) The biofilm biomass was evaluated using the crystal violet binding assay after peptide treatment for 4 h (▽) or 24 h (▵). Percentages were determined relative to the control (untreated biofilm).

4 - Characterization of the cellular density and EPS matrix of *S. aureus* biofilms

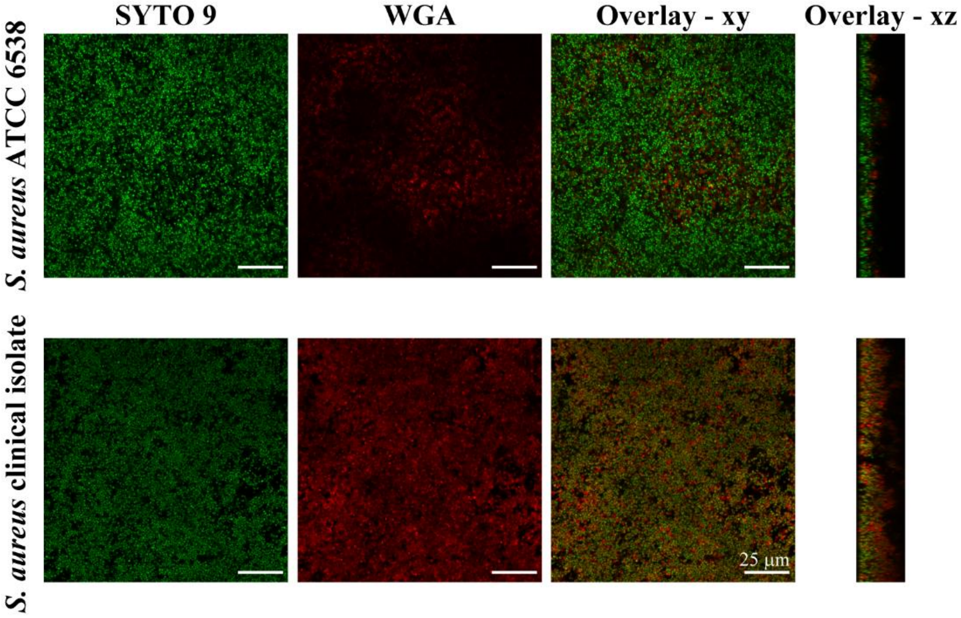

Figure S4 – Bioimaging of biofilms produced by a *S. aureus* reference strain (ATCC 6538) and clinical isolate by CLSM. Biofilms were stained with the nucleic acid stain SYTO 9 (green) and with the sialic acid- and N-acetylglucosamine residues-binding lectin WGA Alexa Fluor 633 (red). The overlay between the green and red channels for the *xy* and *xz* orthogonal plane images are presented. The *xy* plane images were taken at an inner layer of the biofilms (z= 2 µm).

5 - Effect of vCPP2319 redosing on *S. aureus* biofilms

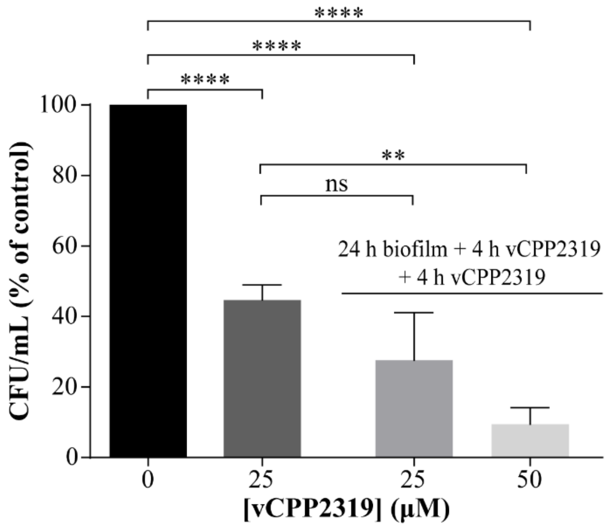

Figure S5 – Effect of vCPP2319 redosing on biofilm-embedded bacteria viability. 24 h-preformed *S. aureus* clinical isolate biofilms were treated with 25 µM vCPP2319 for 4 h. After a washing step with MHB to remove the peptide, biofilms were further treated with 25 or 50 µM of vCPP2319 for an additional period of 4 h. Bacterial viability was evaluated using a colony count assay. Percentages were determined relative to the control (untreated biofilm). ns = non-significant; *p-value ≤ 0.05; **p-value ≤ 0.01; ****p-value ≤ 0.0001.

6 - Antibiofilm properties of α-amylase

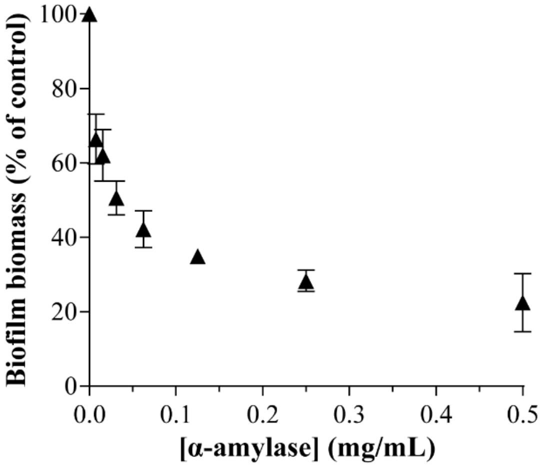

Figure S6 – Effect of α-amylase on biofilm biomass produced by *S. aureus* biofilms. 24 h-preformed *S. aureus* clinical isolate biofilms were treated with variable concentrations of α-amylase for 4 h. The effect on biofilm biomass was evaluated using a crystal violet binding assay. Percentages were determined relative to the control (untreated biofilm).

7 - CLSM Supplementary videos: Combined effect of vCPP2319 with α-amylase

Videos S1-S4 (α-amylase and vCPP2319 sequential treatment):
24 h-preformed *S. aureus* clinical isolate untreated biofilms (S1), or treated with 0,13 mg/mL α-amylase (S2), 25 µM vCPP2319 (S3) or 0.13 mg/mL α-amylase and 25 µM vCPP2319 added sequentially (S4). Biofilms were stained with SYTO 9 and WGA Alexa Fluor 633.
